# The 90% minimum effective concentration of ropivacaine for ultrasound‐guided interscalene brachial plexus block in children aged 1–10 years: A biased coin design up‐and‐down sequential allocation trial

**DOI:** 10.1002/pdi3.82

**Published:** 2024-05-20

**Authors:** Li Yang, Fei Yang, Yaqiong Tian, Ling Liu, Shangyingying Li, Tauseef Ahmad, Yuan Shi, Shengfen Tu

**Affiliations:** ^1^ Department of Anesthesiology Ministry of Education Key Laboratory of Child Development and Disorders National Clinical Research Center for Child Health and Disorders China International Science and Technology Cooperation Base of Child Development and Critical Disorders Children's Hospital of Chongqing Medical University Chongqing China; ^2^ Chongqing Key Laboratory of Pediatrics Chongqing China

**Keywords:** interscalene brachial plexus block, MEC90 (90% minimum effective concentration), pediatrics, ropivacaine, ultrasound‐guided

## Abstract

Ropivacaine is a commonly used local anesthetic for brachial plexus blocks in children, but the minimum effective dose of ropivacaine for interscalene brachial plexus blocks has not been reported. The aim of this study was to determine the 90% minimum effective concentration (MEC90) of ropivacaine for an ultrasound‐guided interscalene brachial plexus block (ISB). A total of 155 patients, aged from 1 to 10 years, underwent unilateral surgical procedures on areas of the upper extremity not innervated by the ulnar nerve. The biased coin design up‐and‐down sequential method (BCD‐UMD) was used to determine the MEC90 of ropivacaine for ultrasound‐guided ISB. In our study, the initial concentration of ropivacaine was 0.07% in the toddler group and 0.09% in the preschool and school‐age groups. During the trial, the concentration of ropivacaine for each subsequent patient was determined by the blocking effect of the previous patient. In case of failure, the concentration for the next patient was increased by 0.01%. Otherwise, the concentration was either decreased by 0.01%, with a probability of 0.11, or kept the same, with a probability of 0.89. Overall, the MEC90 of ropivacaine was 0.104% (95% confidence interval (CI), 0.070%–0.106%) in the toddler group, 0.114% (95% CI, 0.090%–0.117%) in the preschool group, and 0.133% (95% CI, 0.099%–0.136%) in the school‐age group. No adverse events occurred. Our study showed that lower concentrations of ropivacaine could provide effective nerve blocks and reduce the risk of local anesthetics.

## Graphical abstract

The biased coin design up‐and‐down sequential method (BCD‐UMD) was used to determine the MEC90 of ropivacaine for ultrasound‐guided interscalene brachial plexus block. Our study showed that lower concentrations of ropivacaine could provide effective nerve blocks and reduce the risk of local anesthetics.

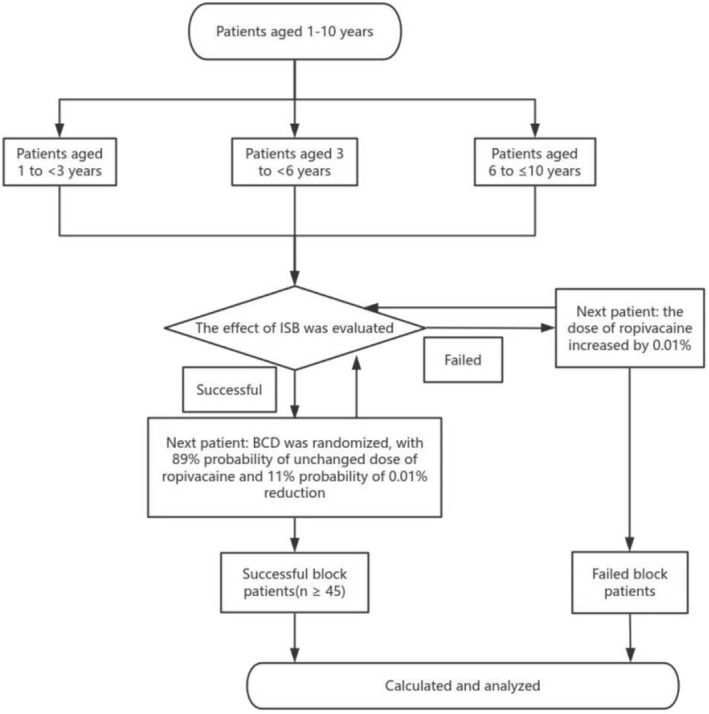

## 1 INTRODUCTION

Ultrasound‐guided brachial plexus block can provide excellent analgesia in children undergoing upper limb surgery. Interscalene brachial plexus block (ISB) is widely used for inducing anesthesia in children undergoing upper limb surgery because of its simple operation and few complications, such as pneumothorax.

Ropivacaine is a new type of long‐acting amide local anesthetic. Compared with other local anesthetics, it has exact anesthetic and analgesic effects but is less toxic to the central nervous system and cardiovascular system.^1^ The volume and concentration of ropivacaine commonly used for brachial plexus blocks in children are 0.5 mL/kg^1^, ^2^, ^3^ and 0.2%–0.5%, respectively.^1^, ^2^, ^3^, ^4^, ^5^ To reduce the adverse effects of local anesthetics, it is important to use an appropriate dose for patients. However, to our knowledge, there are no relevant reports about this topic at present. Therefore, studying the minimum effective dose of local anesthetics is important.

In our study, a prospective sequencing trial was designed to determine the 90% minimum effective concentration (MEC90) of 0.5 mL/kg ropivacaine for ISB in children aged from 1 to 10 years. The aim is to help providing guidance for selecting the best medication in clinical practice.

## 2 MATERIALS AND METHODS

### 2.1 Ethics

The study was approved by the Institutional Ethics Committee of the Children's Hospital of Chongqing Medical University, Chongqing City, China (approval number: [161‐1]/2020, approval date: 12/23/2021). The trial was registered prior to patient enrollment at the Chinese Clinical Trial Registry (ChiCTR2200057830, Principal investigator: Li Yang, Date of registration: 18/03/2022). The parents or guardians of all participants in the study were fully informed of all the experimental processes, the potential benefits and risks, and signed an informed consent form before participation. Patients were enrolled from 18 March 2022, to September 16, 2023.

### 2.2 Participants

Patients who underwent unilateral surgery on the upper extremity without being innervated by the ulnar nerve at the Children's Hospital of Chongqing Medical University were prospectively enrolled. The inclusion criteria were age from 1 to 10 years and an American Society of Anesthesiologists physical status of I‐II. The exclusion criteria were as follows: coagulation dysfunction or receiving anticoagulation treatment, puncture site infection, documented history of local anesthetic hypersensitivity, nerve damage or upper extremity paresthesia, intellectual disability, or declined to participate. The first patient who participated in the study was enrolled on April 15, 2022 and the last patient who participated in the study was enrolled on February 26, 2023. The participants were categorized into three distinct groups based on age: the toddler group (1 to < 3 years old), the preschool group (3 to < 6 years old) and the school‐age group (6 to ≤ 10 years old).

### 2.3 Blinding method

A double‐blind method was employed for the study. An experienced anesthesiologist who was blinded to the method and dosage of ropivacaine performed the ultrasound‐guided ISB. A separate research coordinator, was also blinded to the intervention, collected all the observation data and conducted the postoperative follow‐up examination. Neither the children nor their guardians were aware of the ropivacaine dosage.

### 2.4 Anesthesia procedures

For preoperative preparation, all the children fasted for at least 6 h and were subjected to fluid restriction for at least 2 h prior to surgery. Intravenous access was established in the ward. After entering the operating room, the electrocardiogram, peripheral blood oxygen saturation (SpO2), heart rate (HR), and respiratory rate were continuously monitored, and blood pressure was measured every 5 min. Furthermore, the bispectral index (BIS) was used to monitor the depth of anesthesia. Anesthesia was induced with 0.1 mg/kg of midazolam, 0.2 μg/kg of sufentanil, and 3 mg/kg of propofol. Propofol (5 mg/kg/h) was used to maintain the depth of anesthesia. All children who maintained spontaneous breathing received routine oxygen supplementation via masks at 3 L/min throughout the perioperative period.

**FIGURE 1:**
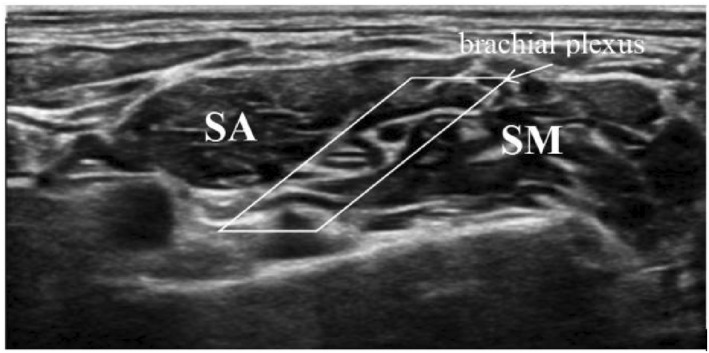
The brachial plexus before injection.

**FIGURE 2:**
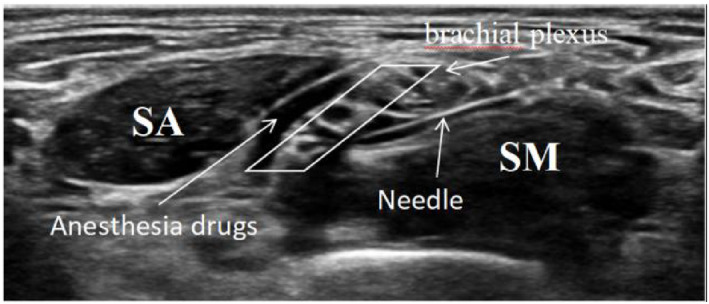
The brachial plexus is soaked with a local anesthetic.

An ultrasound‐guided ISBs was then performed. The patients were placed in the supine position with the arms close to both sides of the body, the face turned to the opposite side of the block, and the neck extended to facilitate probe positioning. The skin was sterilized, and the ultrasound probe (GE venue 50; GE) was wrapped in a sterile sleeve. In the craniocaudal plane, with the ultrasound probe positioned superior to the clavicle, the probe was moved slightly upward until the transverse process of the seventh cervical vertebra was clearly identified. The brachial plexus is located between the anterior scalene muscle and the middle scalene muscle, as shown in Figure 1. The puncture needle was inserted from the outside of the long axis of the ultrasonic probe, and the puncture needle body was kept on the same plane as the long axis of the ultrasonic beam. A small amount of saline (<0.5 mL) was injected when the needle tip reached around the nerve to ensure that the needle tip broke through the fascia, and approximately half of the local anesthetic was injected after pumping back without blood. During drug injection, local anesthetics were injected without resistance to form a dark liquid area around one side of the brachial plexus bundle. Then, the needle was gently retracted, the needle angle was adjusted, the gap between the nerve roots was identified, the needle tip was passed through to the other side of the nerve bundle, and the remainder of the drug was injected. All procedures were performed by the same experienced anesthesiologist. Figure 2 shows the brachial plexus after the block. The skin of the patients with the expected incision site was clamped at 5, 10, 15, and 20 min after the end of the nerve block. Pain is defined as follows:

1. The occurrence of retraction reflex
2. The HR or blood pressure increases by more than 20% compared with that before the nerve block.

The operation was not started until the patient ceased to respond to stimuli.

### 2.5 The biased coin design up‐and‐down sequential method

Dixon's up‐and‐down method^6^ was used to study the median effective concentration of 0.5 mL/kg ropivacaine in our preliminary experiment, and the results were 0.071% (95% confidence interval (CI), 0.058%–0.082%) in the toddler group, 0.089% (95% CI, 0.059%–0.102%) in the preschool group, and 0.091% (95% CI, 0.077%–0.105%) in the school‐age group. According to previous studies,^7^ the volume of ropivacaine in all three groups was 0.5 mL/kg. Based on our pilot trial, we selected 0.07% in the toddler group, 0.09% in the preschool group, and 0.09% in the school‐age group as the first patient’s concentration. The biased coin design up‐and‐down sequential method (BCD‐UDM) was used to study the MEC90. The concentration of ropivacaine for the following participants was determined according to the blocking effect of the previous participant. The concentration gradient for adjacent patients was 0.01%. If the block was successful, the following patients received either the same concentration of ropivacaine (0.89 probability) or a 0.01% reduction in ropivacaine (0.11 probability); this probability value was statistically calculated.^8^ To estimate the MEC90, a minimum of 45 positive responses were required.^9^ When the number of successful block cases reached 45, the trial ended.

### 2.6 Data acquisition

Heart rate and blood pressure at 1 min before skin incision were taken as baseline values.^10^ The success of the block was based on two criteria:

1. No gross purposeful muscular movement after the skin incision.
2. Changes in HR and blood pressure less than 20% of the baseline values before surgical incision.

Two requirements need to be met at the same time.

If either of the following two conditions occurred, the blocking effect was regarded as failure:

1. A withdrawal reflex during skin clamping, 20 min after the completion of the block.
2. Increase in HR or blood pressure more than 20% above baseline values in response to the surgical incision.^11^

For patients who experienced failure, an intravenous injection of 0.1–0.2 μg/kg of sufentanil with or without 1–2 mg/kg of propofol was administered, as appropriate, to achieve surgical analgesia. If the doses of sufentanil and propofol had to be increased to 0.2 μg/kg and 2 mg/kg, the patient experienced no obvious pain relief, or the patient has dyspnea during the operation, general anesthesia had to be induced with a laryngeal mask. After the operation, the infusion of propofol was stopped, and the children were transferred to the postanesthesia care unit (PACU). If the Steward score was ≥4 and the modified Children's Hospital of Eastern Ontario Pain Scale (mCHEOPS) score was <6, the patient was allowed to leave. Postoperative pain was evaluated at 6 and 12 h after the patient returned to the ward, which was completed by the research coordinator using the mCHEOPS score. Postoperative follow‐up to monitor adverse reactions, such as pneumothorax, vascular injury, Horner's syndrome, dyspnea, nerve injury, nausea, and vomiting, was subsequently conducted.

The study coordinator recorded the general condition of the patient, including the patient's age, weight, sex, site of the operation, type of surgery, adverse events, and vital signs during the operation, and recorded the duration of anesthesia (from anesthesia induction to the time of leaving the PACU) and the duration of the operation.

### 2.7 Statistical analysis

In this study, the biased coin design up‐and‐down sequential method (BCD‐UDM) was used. The MEC90 was calculated using isotonic regression. The 95% CIs of the results were calculated by bootstrapping an algorithm with 2000 repeated samples. Further analysis was performed using isotonic regression and bootstrapping CI to calculate the MEC90 for a 99% success rate of block (MEC99).^12^ We used the dose estimator μ3, defined as the interpolated dose that has an estimated probability of effect exactly equal to 0.9 in this study.^13^ Statistical analysis was performed using R3.5.3 (R Foundation for Statistical Computing). The mean ± standard deviation was used to represent the data distribution of continuous variables. The frequency and/or percentage of all categorical variables were calculated.

## 3 RESULTS

**FIGURE 3:**
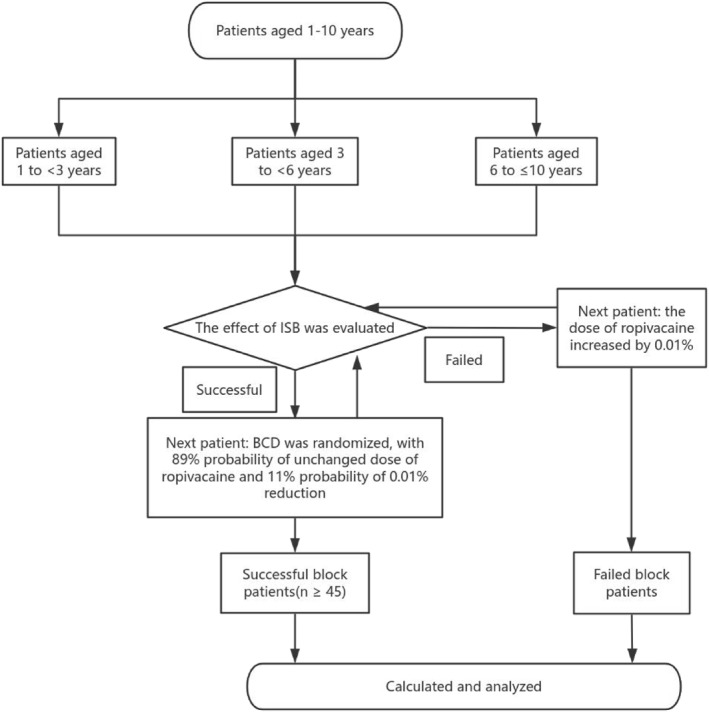
Participant flow of the study.

In total, 155 children completed the study. There were 51 children in the toddler group, 51 in the preschool group, and 53 in the school‐age group. The data of all the patients who met the inclusion criteria and participated in the study were included in the analysis. Figure 3 shows the flow of participant inclusion in the study. No adverse events occurred.

In all the failures, there were six in the infant group, six in the preschool group, and eight in the school‐age group. Block failure during the time of skin incision during surgery. All of them had an increase in HR of more than 20% over the baseline, and body movement occurred during skin incisions. We routinely administered propofol (2 mg/kg) and sufentanil (0.2 μg/kg) to the patients whose block failed; among them, in the infant group, the HR of two patients still exceeded 20% of the baseline after receiving the above doses of drugs, and the anesthesia was changed to general anesthesia with a laryngeal mask. The vital signs of the other two groups approached the baseline level after receiving the above doses of drugs. All patients eventually completed the operation with stable vital signs, and no adverse events occurred.

The demographic data for the three groups are shown in Table 1. There was no significant difference of operation side, operation time, or anesthesia time among the three groups.

**TABLE 1:** Demographic data.

	1 to <3 years old (*n* = 51)	3 to < 6 years old (*n* = 51)	6 to ≤ 10 years old (*n* = 53)
Gender: Male/female(*n*/*n*)	25/26	25/26	39/14
Weight(kg)	11.5 ± 2.5	18.9 ± 4.6	26.6 ± 6.1
Age(months)	20.8 ± 7.7	54.6 ± 10.2	93.2 ± 13.4
Operation side(L/R)	20/31	24/27	24/29
Operation time(min)	48.4 ± 18.6	48.6 ± 26.7	41.3 ± 19.4
Anesthesia time(min)	108.4 ± 24.9	102.2 ± 28.9	98.1 ± 23.2

*Note*: Data are presented as the mean ± SD.

The types of successful and failed surgeries in the three age groups are shown in Table 2.

**TABLE 2:** The type of surgery.

	Successful (45)	Failed (6)	Total (51)
1 to < 3 years old			
Humerus fracture	11	3	14
Congenital thumb polydactyly	31	3	34
Middle finger flexion release	1	0	1
2–3 syndactyly correction surgery	2	0	2
3 to < 6 years old			
Humerus fracture	34	5	39
Congenital thumb polydactyly	6	1	7
Middle finger cyst resection	1	0	1
Broken index finger	1	0	1
Radius fracture	3	0	3

	Successful (45)	Failed (8)	Total (53)
6 to ≤ 10 years old			
Humerus fracture	29	7	36
Congenital thumb polydactyly	10	0	10
Humeral internal fixation removal	1	0	1
Radius fracture	5	1	6

**FIGURE 4:**
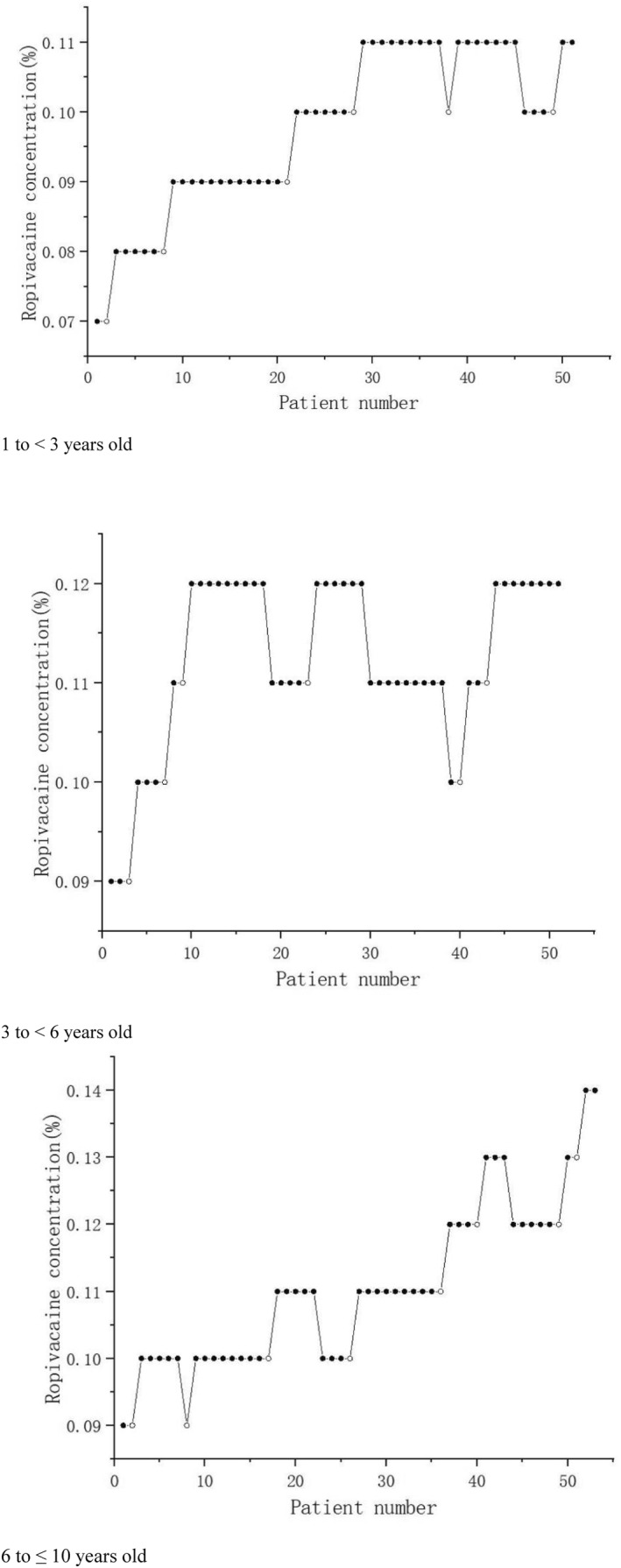
Ropivacaine concentration in each patient with the response (successful concentration is represented by a solid circle; An open circle is used to represent the failure concentration).

The patients' block sequence diagrams of each group are shown in Figure 4.

The MEC90 and MEC99 of 0.5 mL/kg ropivacaine in an ultrasound‐guided ISB were 0.104% (95% CI, 0.070%–0.106%) and 0.109% (95% CI, 0.099%–0.110%) in the toddler group, 0.114% (95% CI, 0.090%–0.117%) and 0.119% (95% CI, 0.110%–0.120%) in the preschool group, and 0.133% (95% CI, 0.099%–0.136%) and 0.139% (95% CI, 0.119%–0.140%) in the school‐age group, respectively.

## 4 DISCUSSION

Age is reportedly an independent predictor of blocking failure.^14^ According to the growth and development characteristics of the children, the patients were divided into a toddler group (1 to < 3 years old), a preschool group (3 to < 6 years old), and a school‐age group (6 to ≤ 10 years old).

In this study, we were the first to report the 90% MEC90 of 0.5 mL/kg ropivacaine for ultrasound‐guided ISB in children of different ages. We used the BCD‐UDM to calculate the MEC90 directly. The high quantile accuracy obtained by the BCD‐UDM used in this study is more accurate than the median effective dose obtained by the Dixon‐Mood up‐and‐down method and is more popular in clinical practice.

A study showed that the size of the peripheral nerve cross‐sectional area increases in an age‐dependent manner in children under 14 years old.^15^ Our preliminary measurements revealed that the diameter of the brachial plexus increases with age. In this regard, we suspect that the older the child is, the larger dose of local anesthetics for nerve block they need. According to our results, among the three age groups, the school‐age group had the highest concentration, while the toddler group had the lowest concentration, which confirms our speculation. Fang G et al.^16^ reported that the MEC90 of 40 mL ropivacaine for ultrasound‐guided supraclavicular brachial plexus block was 0.257% w/v in normal middle‐aged adults. One study^17^ reported that the MEC90 of a 20 mL ropivacaine‐infused ultrasound‐guided axillary brachial plexus block was 4.4 mg/mL. As mentioned earlier, the reason for this difference may be that, on the one hand, the cross‐sectional area of the adult brachial plexus is larger than that of children and more local anesthetics are often required to achieve a satisfactory blocking effect; On the other hand, these are not the minimum effective concentrations for the ISB approach. In children, Zadrazil M et al. reported ultrasound‐guided doses of ropivacaine for brachial plexus blocks of 3.3 mg/kg (0–3 years), 3.2 mg/kg (4‐–6 years), and 2.57 mg/kg (7–10 years),^14^ respectively, while our doses were 0.52 mg/kg (1 to < 3 years old), 0.57 mg/kg (3 to < 6 years old) and 0.67 mg/kg (6 to ≤ 10 years old), respectively. The probable reason was that they did not use the lowest effective dose in the study.

One point to explain is that we did not include surgery in the innervation area of the ulnar nerve for the following reasons: ISB proposed by Winnie in 1970,^18^ is the easiest to learn and master, even in very obese people, and the target structure is easy to find; however, the ulnar nerve mainly originates from the C8 and T1 spinal nerve fibers. Intermuscular sulcus brachial plexus block may be incomplete or delayed in ulnar surgery.

It is worth mentioning that there are considerable individual differences in the growth and development of children, and the volume of ropivacaine is individually administered at mL/kg instead of mL.

In addition, this study has several limitations. First, the duration of surgery in our study was less than 3 hours, and whether the concentration of ropivacaine is suitable for longer surgeries remains to be studied. For ethical reasons, we used sufentanil to reduce puncture pain in children, but we believed that such a small dose had little effect on the results of the study. In the end, our results can only represent brachial plexus block through the intermuscular sulcus approach, and other approaches for brachial plexus block remain to be studied.

In summary, our study demonstrated that the MEC90 of ropivacaine increased with age in children received ISB. Additionally, lower concentrations of ropivacaine could provide dependable analgesia for upper extremity procedures and diminish the likelihood of direct neurotoxicity and systemic toxicity associated with local anesthetics.

## AUTHOR CONTRIBUTIONS

Li Yang helped recruit patients, collect data, analyze and interpret data and write the original draft of the paper. Fei Yang helped design study, recruit patient, collect and analyze data. Yaqiong Tian helped design study, recruit patient, collect and analyze data. Ling Liu helped design study and acquire data. Shangyingying Li helped acquire data. Tauseef Ahmad helped correct the grammatical mistakes. Yuan Shi helped conceive and design the study. Shengfen Tu helped conceive and design, acquire data, revise article, and was responsible for approving the version to be published.

## CONFLICT OF INTEREST STATEMENT

The authors declare no conflicts of interest.

## ETHICS STATEMENT

This study received ethics approval from the Institutional Review Board of the Children's Hospital of Chongqing Medical University (Approval number:[161‐1]/2020, Approval date: 12/23/2021).

## CONSENT FOR PUBLICATION

Not applicable.

## Data Availability

The data that support the findings of this study are available from the corresponding author upon reasonable request.
